# Epithelial differentiation with microlumen formation in meningioma: diagnostic utility of NHERF1/EBP50 immunohistochemistry

**DOI:** 10.18632/oncotarget.25595

**Published:** 2018-06-19

**Authors:** Maria-Magdalena Georgescu, Adriana Olar, Bret C. Mobley, Phyllis L. Faust, Jack M. Raisanen

**Affiliations:** ^1^ Department of Pathology, Louisiana State University and Feist-Weiller Cancer Center, Shreveport, 71103, LA, USA; ^2^ Department of Pathology and Laboratory Medicine and Neurosurgery, Medical University of South Carolina and Hollings Cancer Center, Charleston, 29425, SC, USA; ^3^ Department of Pathology, Vanderbilt University Medical Center, Nashville, 37232, TN, USA; ^4^ Department of Pathology and Cell Biology, Columbia University, New York, 10032, NY, USA; ^5^ Department of Pathology, The University of Texas Southwestern Medical Center, Dallas, 75390, TX, USA

**Keywords:** NHERF1/EBP50, chordoid meningioma, epithelial differentiation, microvilli, NF2

## Abstract

Meningioma is a primary brain tumor arising from the neoplastic transformation of meningothelial cells. Several histological variants of meningioma have been described. Here we show that NHERF1/EBP50, an adaptor protein required for structuring specialized polarized epithelia, can distinguish meningioma variants with epithelial differentiation. NHERF1 decorates the membrane of intracytoplasmic lumens and microlumens in the secretory variant, consistent with a previously described epithelial differentiation of this subtype. NHERF1 also labels microlumens in chordoid meningioma, an epithelial variant not previously known to harbor these structures, and ultrastructural analysis confirmed the presence of microlumens in this variant. NHERF1 associates with the ezrin-radixin-moesin (ERM)-NF2 cytoskeletal proteins, and moesin but not NF2 was detectable in the microlumens. In a meningioma series from 83 patients, NHERF1 revealed microlumens in 87.5% of the chordoid meningioma (*n* = 25) and meningioma with chordoid component (*n* = 7) cases, and in 100% of the secretory meningioma cases (*n* = 12). The most common WHO grade I meningioma variants lacked microlumens. Interestingly, 20% and 66.6% of WHO grades II (*n* = 20) and III (*n* = 3) meningiomas, respectively, showed microlumen-like NHERF1 staining of ultrastructural tight microvillar interdigitations, mainly in rhabdoid, papillary-like or sheeting areas, revealing a new subset of high grade meningiomas with epithelial differentiation. NHERF1 failed to detect microlumens in 12 additional cases of chordoid glioma of the 3rd ventricle, chordoma and chondrosarcoma, neoplasms that may mimic the histological appearance of chordoid meningioma. This study uncovers features of epithelial differentiation in meningioma and proposes NHERF1 immunohistochemistry as a method of discriminating chordoid meningioma from neoplasms with similar appearance.

## INTRODUCTION

Meningiomas are common primary central nervous system (CNS) tumors that derive from meningothelial cells. Thirteen histologic variants of meningioma are recognized in the WHO Classification of Tumors of the CNS [1]. Most of the variants have low recurrence rates, and are therefore considered WHO grade I neoplasms. However, four of these variants are important to distinguish because they portend a worse prognosis: these aggressive variants include chordoid and clear cell meningioma (WHO grade II), as well as papillary and rhabdoid meningioma (WHO grade III).

Neoplastic meningothelial cells may undergo differentiation towards a fibroblastic or an epithelial phenotype. Two histologic variants are currently known to demonstrate epithelial differentiation: secretory and chordoid [2, 3]. Whereas the secretory variant forms intracytoplasmic lumens/inclusions filled with eosinophilic material, the chordoid variant contains cords of cells surrounded by mucoid/myxoid secretions. Moreover, ultrastructural studies have described membranes containing microvilli in both variants [2, 3].

Specialized membranes containing microvilli are usually seen in the polarized cells of epithelia [4]. The microvilli are finger-like projections of plasma membrane with a central actin filament core. The formation of microvilli requires the presence of a structural apparatus formed by proteins that interconnect the actin filaments of the core, connect the actin filaments to the membrane, and in highly organized membranes, such as the brush border membrane of the intestine, connect the microvilli membranes laterally [5]. The ezrin-radixin-moesin (ERM) family of proteins and their associated adaptor protein Na/H exchanger 3 regulatory factor 1/ERM-binding phosphoprotein 50 (NHERF1/EBP50) are involved in maintaining the bond between the actin filament core and the membrane of the microvillus [4], and NHERF1 is required for the process of microvillus formation and organization *in vitro* and *in vivo* [6, 7]. Consistent with its role in microvillus morphogenesis, NHERF1 has been shown to be a very sensitive marker of polarity structures containing microvilli, such as microlumens, and a clinical assay for NHERF1 expression is currently used to improve the diagnostic accuracy of microlumen-containing brain tumors [8, 9]. In addition, NHERF1 expression in various subcellular compartments is clinically used for breast cancer patient prognostic stratification [10].

Here, we explored epithelial differentiation in meningioma by using NHERF1 to detect polarity structures. We confirmed that secretory meningioma contains intracytoplasmic lumens/inclusions and microlumens, and additionally found that chordoid meningioma contains NHERF1-labeled microlumens that were further characterized ultrastructurally. By analyzing a panel of 95 brain tumors, including tumors from the differential diagnosis of chordoid meningioma, we propose NHERF1 as an immunohistochemical diagnostic marker for microlumen or microlumen-like structure detection in secretory, chordoid and a new subset of high grade meningioma with rhabdoid, papillary-like or sheeting morphology.

## RESULTS

### NHERF1 detects intracytoplasmic inclusions and microlumens in secretory meningioma

**Figure 1:**
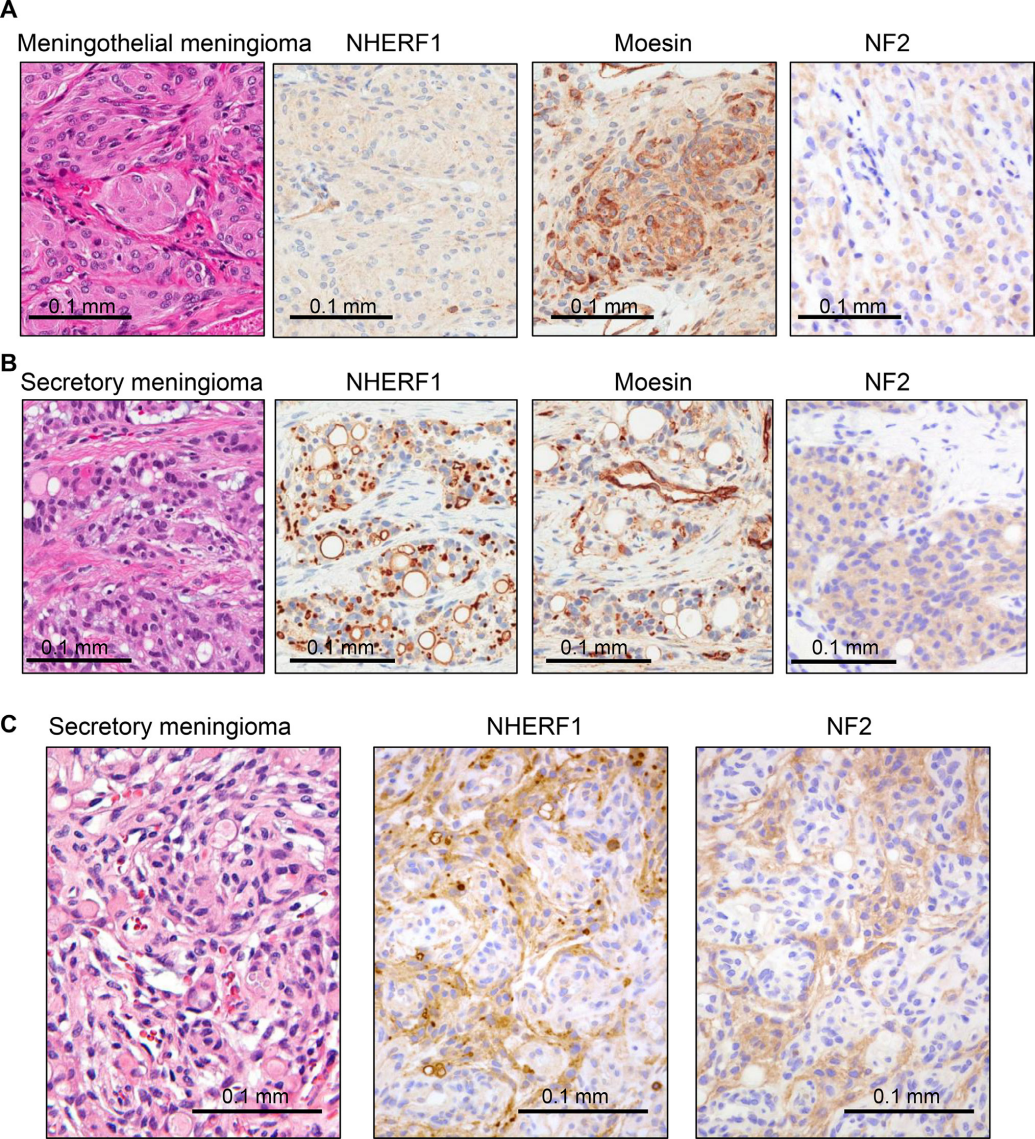
NHERF1, moesin and NF2 expression in WHO grade I meningioma (**A**) IHC with NHERF1, moesin and NF2 antibodies in meningothelial meningioma shows cytoplasmic distribution for all three proteins. (**B**) The expression of the same proteins in secretory meningioma shows labeling of intracytoplasmic lumens and of microlumens by NHERF1 and moesin, and strong cytoplasmic expression of NF2. (**C**) In secretory meningioma with a whorling meningothelial component, there is strong differential expression of NHERF1 and NF2, including the presence of NHERF1-labeled microlumens, in the secretory component.

To explore epithelial differentiation in meningioma, we used NHERF1 antibody to immunolabel several variants of meningioma. The meningothelial variant showed weak cytoplasmic NHERF1 staining (Figure 1A), similar to other common variants, such as fibrous, transitional and psammomatous meningioma. In contrast, NHERF1 labeled variably intracytoplasmic lumens/inclusions and revealed a multitude of microlumens in the secretory variant (Figure 1B). Moesin but not the related ERM family member merlin, the product of the neurofibromin 2 (NF2) gene, hereafter called NF2, has been shown to colocalize with NHERF1 in the microlumens of ependymoma [9]. In meningioma, moesin labeled strongly the vessels and to a lesser extent the lumens and microlumens in the secretory variant (Figure 1A–1B) and NF2 labeled the cytoplasm, more intensely in the secretory variant (Figure 1A–1B). Interestingly, both NF2 and NHERF1 showed higher cytoplasmic expression in the secretory component of mixed secretory-meningothelial meningioma (Figure 1C).

**Figure 2:**
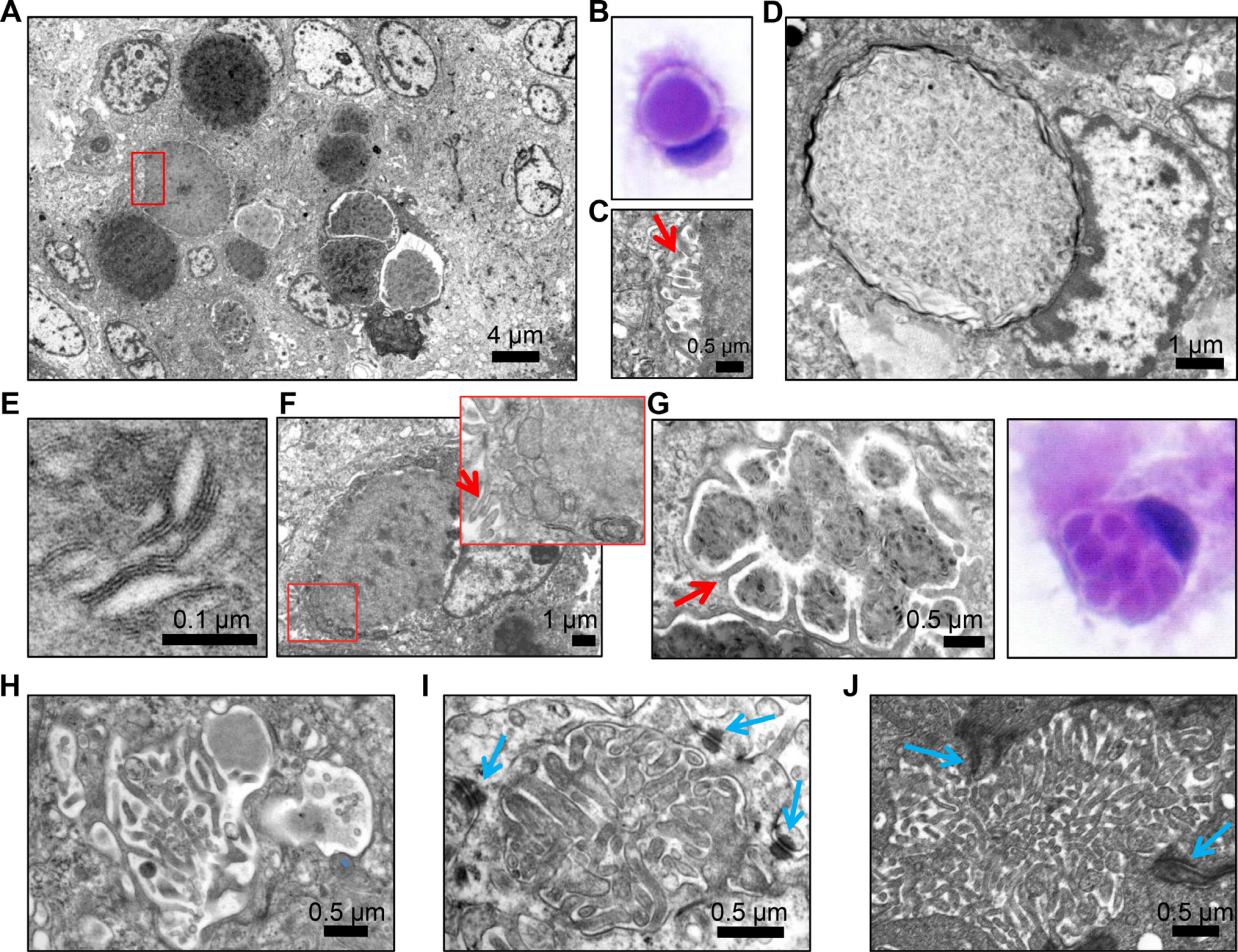
Transmission electron microscopy (TEM) demonstrates microvilli-containing lumens and microlumens in secretory meningioma (**A**) Low magnification view of secretory meningioma showing a cluster of intracytoplasmic lumens with various compaction of the content. (**B**) H&E cytologic preparation of secretory meningioma showing a cell with an intracytoplasmic lumen/inclusion pushing the nucleus. Note the clear space between the content and the periphery of the inclusion. (**C**) High magnification of the boxed area in (A) showing that the clear space from (B) contains microvilli (arrow). (**D**) Intracytoplasmic lumen/inclusion with blunted microvilli-devoid surrounding membrane. (**E**) High magnification of the lamellar content of inclusions containing compacted material. (**F**) Cell with intracytoplasmic inclusion and tightly apposed mitochondria and rough endoplasmic reticulum (inset) and microvilli (arrows) on the plasma membrane. (**G**–**H**) Ultrastructural and H&E cytologic appearance of intracytoplasmic multiloculated inclusions compartmentalized by long microvilli (arrow in G). (**I**) Microlumen with dense, thick microvilli containing an actin filament core. Blue arrows indicate surrounding desmosomes. (**J**) Comparative view of a microlumen from ependymoma showing abundant microvilli within a lumen delimited by adherens junctions (arrows).

Ultrastructural analysis of a secretory meningioma revealed electron dense secretions compacted within membrane-delimited vesicles (Figure 2A). These vesicles were intracytoplasmic, as also noted in the cytologic preparation (Figure 2B). These intracytoplasmic vesicles have been previously reported in secretory meningioma, as well as in other pathologic conditions, and they are termed intracytoplasmic lumens or inclusions [2, 11–13]. The membrane of some but not all the inclusions formed microvilli at the interface with the content (Figure 2C, microvilli shown by red arrow; Figure 2D, smooth membrane). The content appeared to have variable degrees of compaction and was composed of amorphous proteinaceous material but also by lamellated linear or circular membranous structures (Figure 2D–2E). Mitochondria and rough endoplasmic reticulum were frequently tightly apposed to the inclusions, and microvilli formation was sometimes seen at the periphery of the cell (Figure 2F). Compartmentalization of the inclusions by long microvilli was seen (Figure 2G–2H). Structures closely resembling microlumens surrounded by cell-cell junctions were more rarely seen (Figure 2I, blue arrows label desmosomes). For comparison, an ependymoma microlumen delimited by cell-cell junctions (zonula adherens) is shown (Figure 2J). The structures containing microvilli are most likely responsible for the NHERF1 labeling observed in secretory meningioma.

### NHERF1 reveals microlumens in chordoid meningioma

**Figure 3:**
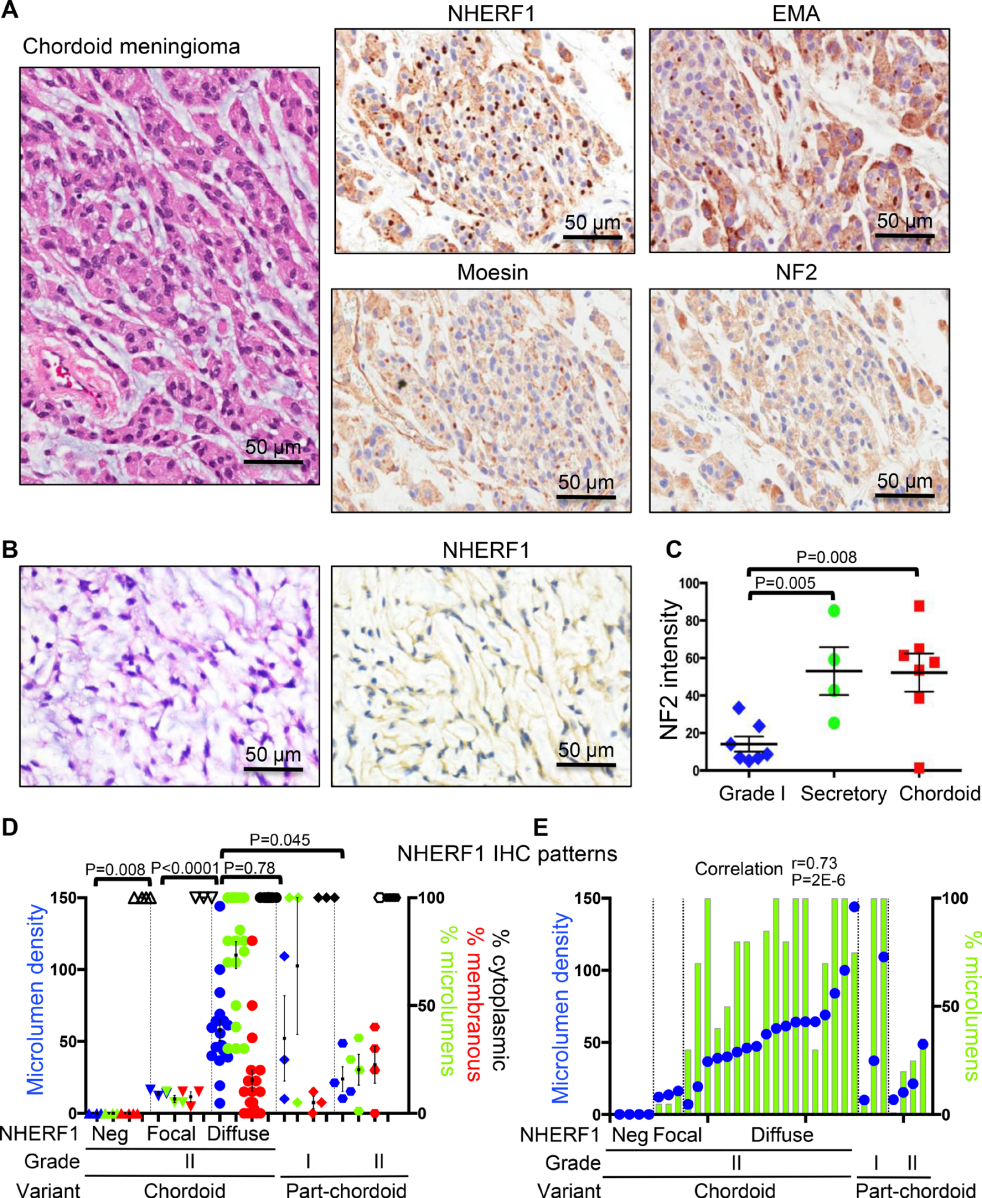
NHERF1, moesin and NF2 expression in chordoid meningioma (**A**) IHC with NHERF1, moesin, NF2 and EMA antibodies in chordoid meningioma shows labeling of microlumens by NHERF1 and to a lower extent by moesin and EMA. NF2 displays strong cytoplasmic expression. (**B**) In a minority of chordoid meningioma cases, NHERF1 expression is limited to the cytoplasm only. (**C**) Quantification of NF2 expression in secretory (*n* = 4) and chordoid (*n* = 7) meningioma as described in Materials and Methods, in comparison to WHO grade I meningioma cases with common meningothelial (*n* = 3), transitional (*n* = 2), fibrous (*n* = 1), and psammomatous (*n* = 1) morphologies. All the samples were run in one experiment to allow comparison. This staining was repeated three times on additional samples and showed similar results. A positive control represented by choroid plexus papilloma [8] was included in each series. (**D**) NHERF1 expression patterns in chordoid and part-chordoid meningioma cases represented as mean ± SEM of individual values. Each value corresponds to a case. The microlumen density, expressed as mean of counts in 3 HPFs, is plotted on the left Y axis, and the tumor areas of the three patterns—microlumens, membranous, cytoplasmic—expressed in %, are plotted on the right Y axis. Open and filled symbols for the cytoplasmic pattern correspond to weak or strong NHERF1 expression, respectively. (**E**) Contingency graph showing significant correlation between microlumen density and % microlumen area, plotted as in (D), in individual cases of chordoid and part-chordoid meningioma.

NHERF1 immunostaining of chordoid meningioma showed a robust dot-like pattern (Figure 3A) in the majority of cases, similar to that observed in ependymoma [9]. Some of the dots were visible by EMA IHC, but their detection was obscured by strong EMA cytoplasmic labeling. As in secretory meningioma, moesin but not NF2 appeared to be expressed with NHERF1 in the dot-like structures (Figure 3A). In a minority of cases, only cytoplasmic NHERF1 staining was seen and the dot-like pattern was absent (Figure 3B). Most of these cases contained fibroblast-like cells, usually in single file, within an abundant myxoid extracellular matrix. NF2 was usually highly expressed in the cytoplasm of neoplastic cells in both secretory and chordoid meningioma (Figures 1B and 3A). Its expression levels were similar in chordoid and secretory meningioma, more than 3-fold higher than in common WHO grade I meningioma variants (Figure 3C). One case of multiply recurrent chordoid meningioma from a patient who succumbed to disease completely lacked NF2 expression (Figure 3C). This tumor also showed only focal NHERF1 dot-like expression.

Chordoid meningioma was diagnosed when more than 50% of the tumor displayed chordoid architecture [14]. Tumors with partial (less than 50%) chordoid architecture were classified as WHO grade I if atypical features or brain invasion were not present, or as WHO grade II when these features were present (Table 1). For the chordoid and part-chordoid meningiomas, we noted three patterns of NHERF1 expression: cytoplasmic, dot/microlumen-like and membranous (Figure 3D). Nuclear expression was not observed in any meningioma histologic variant, in contrast to breast cancer [15, 16]. The quantification of these three IHC patterns as percentage of tumor, as well as of the density of NHERF1 dot/microlumen-like structures, showed significant correlation between the extent of dot/microlumen-like pattern and the microlumen density (*r* = 0.73, *p* = 0.000002; Figure 3E), and resulted in the classification of chordoid meningiomas in three categories (Figure 3D–3E). The NHERF1-negative tumors (*n* = 4) had only weak cytoplasmic expression similar to grade I meningiomas and mostly fibroblastic appearance (Figure 3B). The NHERF1-focal positive tumors (*n* = 3) had weak cytoplasmic expression, some membranous labeling and less or equal to 10% dot-like areas with low microlumen counts. The NHERF1-diffuse positive tumors (*n* = 18) were significantly different from the previous categories by having strong cytoplasmic expression, extensive dot-like areas with high microlumen counts and only occasional membranous expression. In the part-chordoid category, two of the three grade I tumors had similar patterns as the NHERF1-diffuse positive chordoid meningiomas, and the third had focal pattern (Figure 3D–3E and Table 1). It is noteworthy that for the two NHERF1-diffuse positive grade I tumors, the slide used for NHERF1 assessment had 100% chordoid morphology, whereas the grading was performed on the entire available specimen which was short of the 50% cut-off chordoid morphology. These findings might warrant closer follow-up of these patients. The four cases of grade II atypical meningioma with focal part-chordoid morphology had a significantly different overall NHERF1 expression pattern from the NHERF1-diffuse positive chordoid meningiomas (Figure 3D, Supplementary Figure 1 and Supplementary Table 1) and resembled the cases of atypical meningioma with NHERF1 dot-like areas (see below).

**Table 1:** NHERF1 IHC patterns in meningioma and other chordoid/myxoid tumors

Diagnosis	WHO	Patients		Tumor location				NHERF1 IHC	
	grade	No. cases Gender	Median age (range, yrs)	NSB	SB	SC	V	Focal^1^	Diffuse^1^
								(% + cases)	
Grade I^2^ Mng	I	13 (11F; 2M)	49 (33–85)	8	2	3	0	0	0
Secretory^3^ Mng	I	12 (9F; 3M)	55.5 (39–69)	5	7	0	0	8	92
Part chordoid Mng^4^	I II	3 (3F; 0M) 4 (0F; 4M)	60 (51–68) 58.5 (35–78)	0 1	3 2	0 0	0 1	33 25	67 75
Chordoid Mng	II	25 (17F; 8M)	45 (29–87)	12	13	0	0	12	72
Atypical Mng	II	20 (12F; 8M)	59.5 (31–84)	13	7	0	0	10	10
Anaplastic Mng	III	3 (2F; 1M)	44 (30–58)	2	1	0	0	0	67
Clear Cell Mng	II	3 (2F; 1M)	44 (14, 67)	1	1	0	1	33	67
Chordoma		8 (6F; 2M)	50.5 (15–65)	0	6	2	0	Membrane 62%	
Chordoid glioma	II	2 (2M)	43 (40, 46)	0	0	0	2	Membrane 50%	
Chondrosarcoma	I-II	2 (1F; 1M)	47 (32, 62)	0	2	0	0	Negative	

Abbreviations: NSB, non-skull base (hemisphere convexity/falx); SB, skull base; SC, spinal cord; V, ventricular; M, male; F, female; Mng, meningioma.

^1^Focal and diffuse refer to the pattern of NHERF1 microlumen staining on a representative section.

^2^Grade I meningiomas listed here include the following variants: meningothelial (*n* = 5), transitional (*n* = 3), fibrous (*n* = 2), psammomatous (*n* = 2), microcystic (*n* = 1).

^3^One of the 12 secretory meningioma cases was diagnosed as WHO grade II based on increased mitotic activity.

^4^Part chordoid meningioma cases contained less than 50% chordoid histology in the entire tumor.

**Figure 4:**
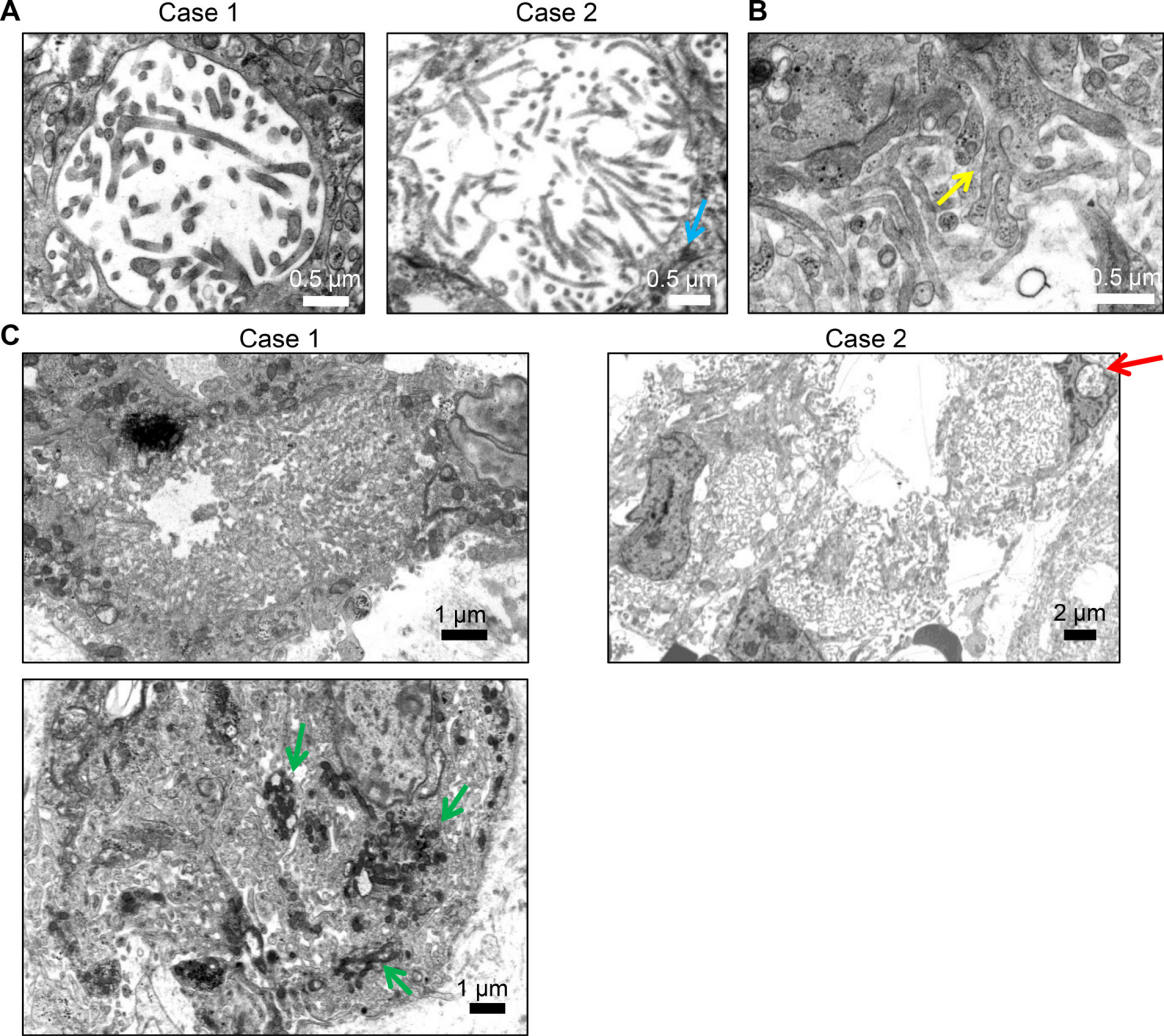
TEM demonstrates microlumens and extensive membrane specialization in chordoid meningioma (**A**) TEM was performed on 2 cases of chordoid meningioma, either subjected fresh to glutaraldehyde fixation (Case 1) or post deparaffination (Case 2). Microlumens containing thin microvilli with an actin filament core were detected in both cases. (**B**–**C**) Extensive lace-like specialized membrane areas were present in both cases, containing both thin microvilli and branched membrane protrusions without filamentous core (B, yellow arrow). Mitochondria may be numerous (green arrows).

To confirm that the NHERF1-positive dot-like structures represented microlumens, we performed an ultrastructural analysis of two representative NHERF1-diffuse positive chordoid meningioma cases. In both instances, we detected microlumens containing thin and long microvillus projections and surrounding desmosome-like junctions (Figure 4A, blue arrow labels desmosome). In addition to microlumens, both cases showed numerous larger membrane lumens filled with microvilli containing an actin microfilament core but also with thicker membrane protrusions, sometimes showing branching (Figure 4B–4C). Numerous mitochondria were seen adjacent to these lumens, suggesting intense secretory activity (Figure 4C, green arrow). The microvillar lace-like areas were present in both cases of chordoid meningioma and were not seen in secretory meningioma. Their presence might explain the robust NHERF1 staining in chordoid meningioma. Nuclear pseudoinclusions are a hallmark of meningioma. Interestingly, these were found in both chordoid and secretory meningioma and contained microvilli or vesicles with secretions, respectively (Figure 4C, red arrow, and Supplementary Figure 2).

The population of patients with chordoid meningioma that we analyzed showed recurrence-free survival of 74.4% at 5 years and of 53.1% at 10 years (Supplementary Figure 3A), values comparable to those from WHO grade II meningioma with other morphologies [1]. A correlation between NHERF1 expression and the recurrence rate could not be determined, as two of the four NHERF1-negative cases with follow-up available did not show recurrences at 2 and 9 years of follow-up. Initial resection and subsequent resection specimens were available in four cases. The recurrent tumors showed chordoid morphology in three cases, while meningothelial morphology dominated the recurrence specimen for the multiply recurrent focal NHERF1-positive NF2-negative case (described above). In all three chordoid recurrent cases, although the histology was epitheliod as in the initial resections, the NHERF1 microlumen pattern was decreased and the membranous pattern was strikingly increased (Supplementary Figure 3B).

### Microlumen distribution in meningioma

**Figure 5:**
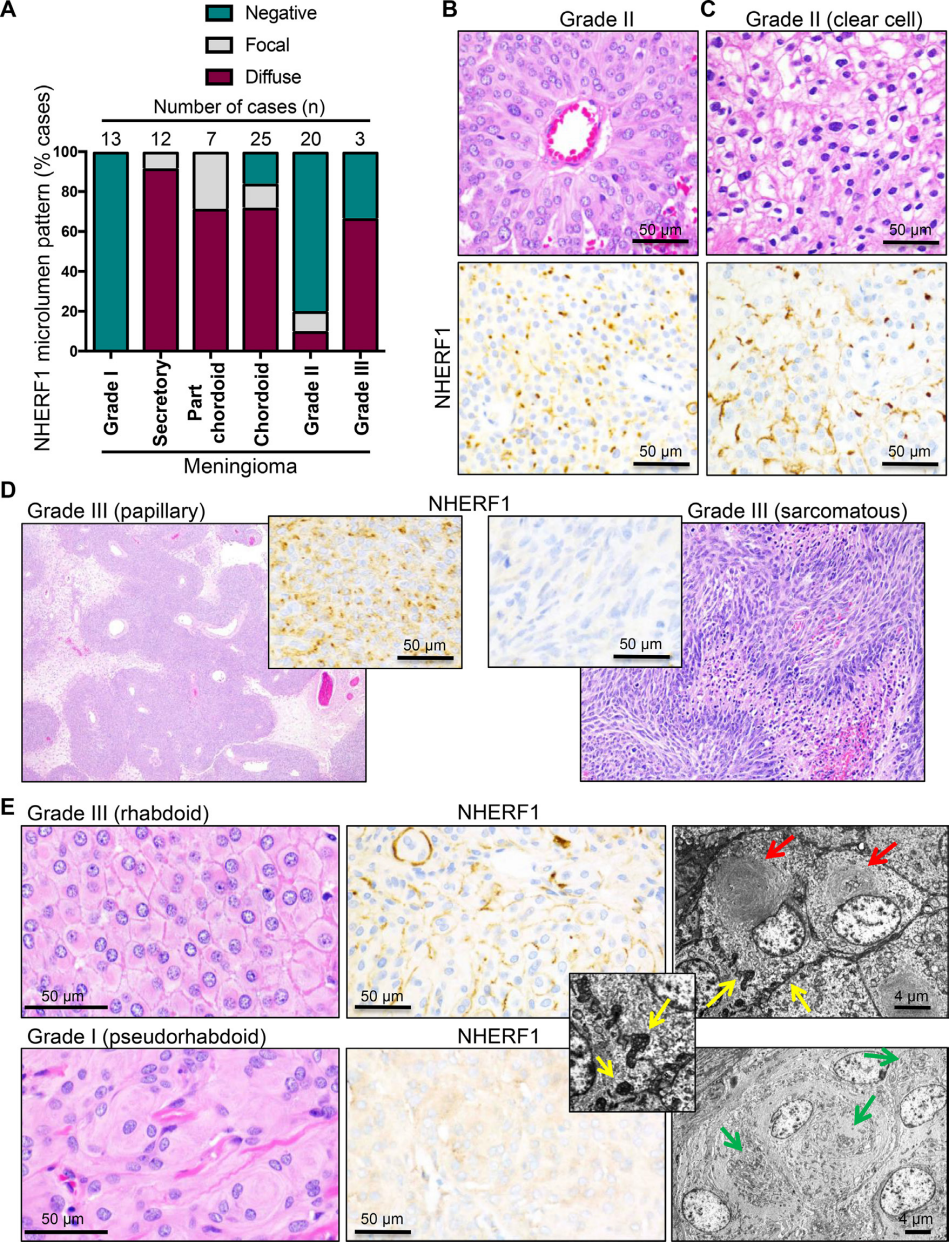
NHERF1 labels microlumens in secretory and chordoid meningioma and tight microvilli-like interdigitations in clear cell, rhabdoid and papillary/papillary-like meningioma (**A**) The distribution of microlumens in meningioma is represented with data selected from Table 1. (**B**) H&E and NHERF1 IHC of the WHO grade II meningioma case with the strongest and most diffuse NHERF1 microlumen labeling. The tumor showed pseudorosetting and sheeting morphology. (**C**) H&E and NHERF1 IHC of a case of clear cell meningioma shows a mixed membraneous and microlumen-like pattern of NHERF1 staining. (**D**) H&E and NHERF1 IHC of WHO grade III meningioma cases shows diffuse NHERF1 microlumen-like labeling in the cases with papillary morphology and lack of labeling in a case with sarcomatous morphology. (**E**) H&E, NHERF1 IHC and TEM in a true rhabdoid area of a complex case of atypical meningioma in comparison with a pseudorhabdoid WHO grade I meningioma case. Red and green arrows show true rhabdoid and pseudorhabdoid inclusions, respectively, and yellow arrows show tight microvilli-like plasma membrane interdigitations, sometimes with grape-like appearance.

To explore epithelial differentiation in meningioma, we labeled with NHERF1 antibody 83 cases of meningioma including the most common variants and all three WHO grades. The demographic data for these patients and the location of the tumors are shown in Table 1. The most common WHO grade I variants did not show microlumen formation (Table 1 and Figure 5A). All 12 cases of secretory meningioma tested were positive for microlumen formation, the vast majority showing a diffuse pattern of immunoreactivity. In a large cohort of 25 chordoid meningiomas, 72% and 12% of the tumors showed diffuse or focal NHERF1 microlumen labeling, respectively (Table 1, Figure 3D and Figure 5A). Since chordoid meningiomas are WHO grade II tumors, we analyzed a prospective cohort of 20 WHO grade II tumors of various morphologic patterns, and 3 cases of clear cell meningioma, a WHO grade II histologic variant. Of the 20 atypical meningioma cases, 4 (20%) were found to display either focal (*n* = 2) or more diffuse (*n* = 2) NHERF1 microlumen staining, as well as membranous expression (Table 1, Supplementary Table 1 and Supplementary Figure 1), whereas the reminder (80%) were negative. The tumors with diffuse NHERF1 microlumen pattern showed sheeting with or without pseudorosetting (Figure 5B). The three cases of clear cell meningioma had a mixed membranous and dot-like pattern of NHERF1 expression, with extensive membranous component (Figure 5C, Table 1, Supplementary Figure 1). Two (75%) out of three WHO grade III tumors were NHERF1 microlumen-positive (Table 1, Supplementary Figure 1, Supplementary Table 1), one showing papillary morphology (Figure 5D, left panels), and one, sheeting. The NHERF1-negative case displayed sarcomatous morphology (Figure 5D, right panels) and completely lacked NHERF1 expression (Supplementary Table 1).

A very rare WHO grade III histologic variant is rhabdoid meningioma. Recently, a study analyzing the patient outcome in a relatively large cohort of meningiomas with variable rhabdoid component has recommended the grading of these tumors analogously to non-rhabdoid tumors [17]. In addition, the authors found that some of these tumors have pseudo-rhabdoid inclusions consisting of accumulations of interdigitating cell processes rather than intermediate filaments [18]. In our series, we had one grade II atypical case with part-rhabdoid, part-chordoid and also sheeting complex morphology that showed the highest extent of both microlumen and membranous NHERF1 labeling within the category of WHO grade II part-chordoid cases (case 32 in Supplementary Table 1). We confirmed true rhabdoid morphology ultrastructurally with bone fide paranulcear shorls of intermediate filaments and showed that in rhabdoid areas NHERF1 has a composite membranous and dot-like expression pattern (Figure 5E upper panels). In contrast a pseudo-rhabdoid case with paranuclear inclusions formed by membrane interdigitations had cytoplasmic NHERF1 staining (Figure 5E, lower panels).

A closer examination of the plasma membrane in the true rhabdoid areas showed crisp membrane boundaries in the H&E preparation, similar to the clear cell and sheeting areas, and in contrast to the syncytial appearance from meningothelial meningioma (compare H&E panels in Figure 5B, 5C and 5E). Ultrastructurally, these prominent cell boundaries in rhabdoid and sheeting areas contained short interdigitating microvilli-like projections that sometimes formed grape-like membrane structures protruding into the cytoplasm (Figure 5E-inset and Supplementary Figure 4). Most likely, these tight microvilli-like interdigitations rendering a zipper-like appearance to the plasma membrane correspond to the NHERF1 membranous staining, and the grape-like intracytoplasmic structures rather than true microlumens, to the NHERF1 dot-like pattern, in high-grade meningiomas with sheeting, clear cell, papillary or rhabdoid change.

### NHERF1 labels the plasma membrane in subsets of chordoid glioma and chordoma

**Figure 6:**
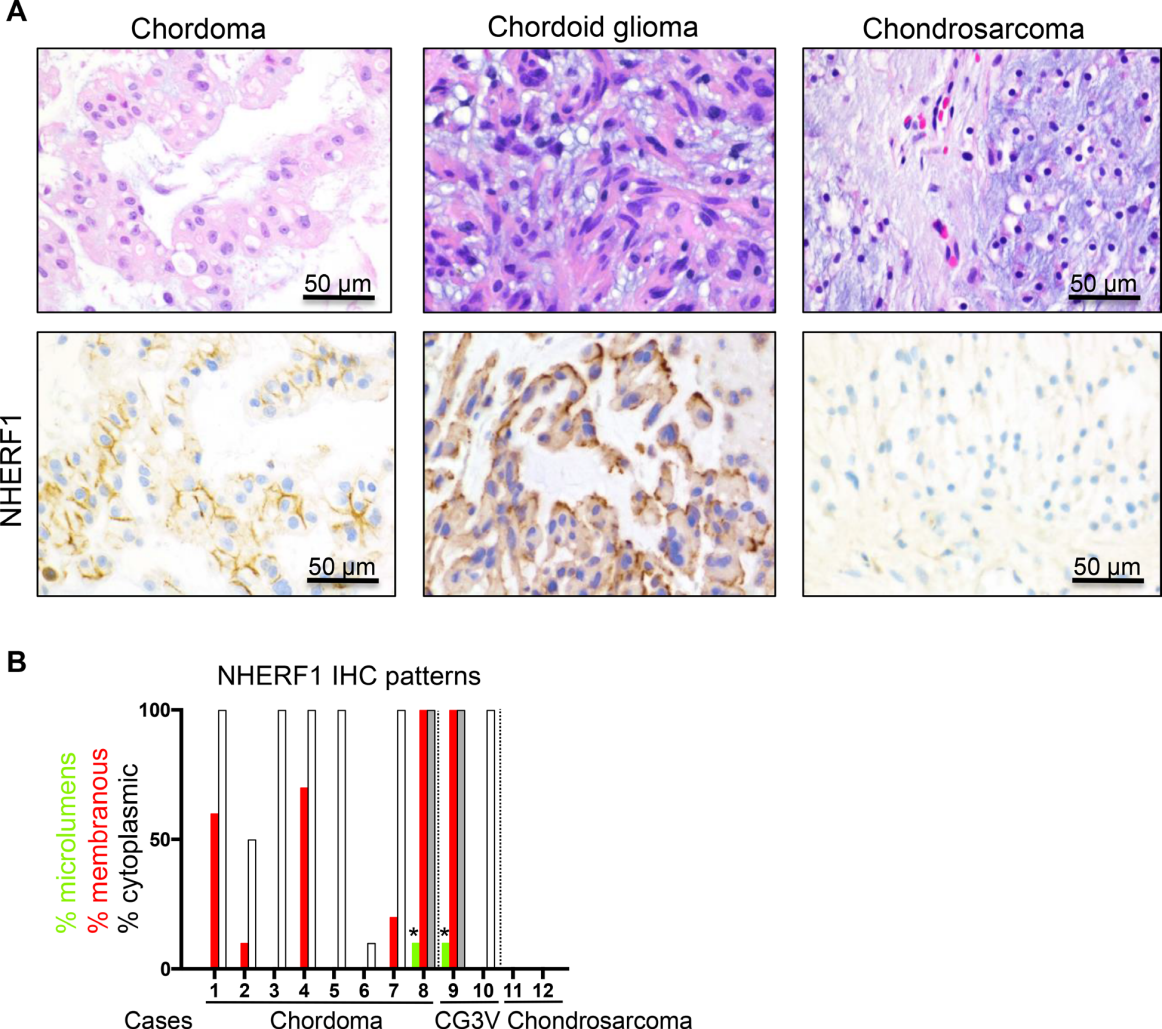
NHERF1 labels the membrane in a subset of chordoma and chordoid glioma and is negative in chondrosarcoma (**A**) NHERF1 IHC in selected examples shows NHERF1 labeling of membrane in chordoma and chordoid glioma. (**B**) Contingency graph showing the distribution of the NHERF1 expression patterns in individual cases. Open and grey bars denote weak or moderate cytoplasmic expression, respectively. The asterisks indicate focal dot-like pattern from membrane fragmentation. CG3V, chordoid glioma of the 3rd ventricle.

To assess the diagnostic utility of NHERF1 in meningioma, specifically for the chordoid variant, we explored the NHERF1 labeling patterns in a panel of 12 tumors with similar morphological appearance, including cases of chordoma, chordoid glioma of the 3rd ventricle and chondrosarcoma [19] (Table 1). From the 8 chordomas tested, 5 showed membranous staining with variable intensity, and 3 were negative (Figure 6). In contrast to chordoid meningioma, the cytoplasmic staining was weak in all cases, except for one case with moderate staining (Figure 6A). One chordoid glioma of the 3rd ventricle showed strong membranous staining at the interface with the extracellular myxoid matrix and one was negative (Figure 6). The two cases of chondrosarcoma tested were negative (Figure 6). None of these chordoid meningioma mimics showed a robust dot-like pattern indicative of microlumens, and in the two cases where a focal dots were present, the pattern was due to membrane fragmentation (Figure 6B). Whereas the NHERF1 membranous staining might suggest the presence of membrane microvilli in these tumor cells, this assumption needs to be confirmed by ultrastructural studies.

## DISCUSSION

Meningiomas are heterogenous tumors and their pathologic classification comprises 13 recognized histologic variants [1], surpassing by far the number of histologic variants of any other primary brain tumor. In addition to intertumoral heterogeneity, intratumoral variation frequently occurs, with combinations of histologic variants seen in individual neoplasms. The pathogenesis of the tremendous variability in meningioma morphology is intriguing and different mechanisms have been proposed, including the embryologic derivation of the cells of origin of these neoplasms [20]. The plasticity of differentiation of these cells, currently termed cancer stem cells, is indeed overwhelming, with two major avenues that have been recognized early in their characterization: the mesenchymal and the epithelial pathways [20]. However, from both a cytomorphologic and a derivation marker standpoint, there are overlaps among all forms of meningioma. For example, ultrastructurally, meningiomas as a whole are characterized by the presence of robust cell-cell junctions, which represent a feature of epithelial differentiation gradually lost in the process of epithelial to mesenchymal transition (EMT) [21–23]. A more specific ultrastructural finding, occurring mostly in epithelial-like variants, is the presence of microvilli in secretory and chordoid meningiomas [2, 3]. From an immunohistochemical marker standpoint, meningiomas are labeled by EMA, a marker of epithelial differentiation. In addition, cytokeratin and vimentin intermediate filaments are present in many of the histologic variants of meningioma.

Since meningioma morphology correlates with outcome, it is important to accurately characterize meningiomas and therefore, the pursuit of more specific markers of meningioma differentiation is worthwhile. In this study, we screened a large array of histologic variants of meningioma for microvillus formation using NHERF1, a marker that labels microvilli from diverse tissues [7, 9, 24, 25]. We found that secretory meningioma contains intracytoplasmic inclusions/lumens and microlumens labeled by NHERF1, consistent with the established epithelial differentiation of this variant. In addition, we discovered that chordoid meningioma displays microlumens strongly labeled by NHERF1, consistent with the epithelial differentiation in this histologic variant. NHERF1 did not detect microlumens in the most common variants of meningioma, including meningothelial, fibrous, transitional and psammomatous. Surprisingly, the NHERF1 labeling pattern was suggestive of the presence of microlumens in a subset of WHO grade II and grade III meningiomas demonstrating clear cell, rhabdoid, papillary, pseudopapillary or sheeting morphology. Electron microscopy showed that rather than true microlumens, the NHERF1 staining in these areas is due to the presence of zipper-like membranes and intracytoplasmic membrane-derived grape-like structures formed by tightly interdigitating short microvilli-like plasma membrane projections. The clue for differentiating these microvilli-like structures from the true microlumens by NHERF1 IHC is their association with membranous staining in the absence of a strong cytoplasmic staining. Indeed, the opposite NHERF1 IHC pattern, i.e. lack of membranous staining and high cytoplasmic expression, is noted in association with microlumens in most cases of chordoid or secretory meningioma. These data suggest that NHERF1 is a reliable marker for epithelial differentiation in meningioma. They also point to the presence of a previously unknown subset of high grade meningiomas with epithelial differentiation.

The program of differentiation of stem cells into specialized epithelia is coordinated by transcription factors that direct the synthesis of proteins involved in polarity [26]. One such polarity protein is NHERF1, which through interactions with the ERM family cytoskeletal proteins, is responsible for epithelial morphogenesis, including microvillus and lumen formation [7, 27, 28]. Due to this key structural role, the expression of NHERF1 in conjunction with ERM proteins would be expected in tumors with epithelial differentiation, including the meningioma variants harboring this differentiation. Indeed, both NHERF1 and NF2 were highly expressed in both secretory and chordoid variants. Interestingly, in secretory meningioma, a specific genetic signature with simultaneous mutations in the KLF4 transcription factor and the TRAF7 E3 ubiquitin ligase has been found [29, 30]. NF2 mutations are absent in this variant, [31] which may explain the high NF2 expressions levels described by us and by others [32]. Although the role of KLF4 in the differentiation of meningioma is not known, it would be intriguing to explore if it controls the transcription of NHERF1, ERMs or other proteins involved in epithelial polarity. Beside the presence of estrogen-responsive elements in the *NHERF1* gene promoter that activate a robust expression of NHERF1 [33], not much is known about the transcriptional regulation of NHERF1. Whether KLF4 and/or the progesterone receptor that appears to be highly expressed in secretory and chordoid meningioma (MM Georgescu, unpublished observations) play a role in NHERF1 upregulation remains to be investigated. Along the same lines, future studies should address the genetic signature of chordoid and papillary meningioma, and if there are common pathways upregulated in these variants that lead to epithelial differentiation.

Consistent with the role of NHERF1 in epithelial differentiation with its loss shown to induce EMT [28], we found lower overall NHERF1 expression in a subset of chordoid meningiomas displaying fibroblastic appearance and the high-grade sarcomatous forms or areas completely lacked NHERF1 expression. In contrast to carcinomas, where EMT is often a morphological feature conferring a worse prognosis [34], the role of EMT in meningioma patient outcome has not been studied. In our series, we did not observe a recurrence predilection for the chordoid meningioma cases with fibroblastic appearance (Supplementary Figure 3).

In this study, we also explored the use of NHERF1 as potential diagnostic marker for chordoid meningioma. Chordoid meningioma is frequently located at the skull base where other extraaxial chordoid or mucinous neoplasms may arise, including chordoma and chondrosarcoma. Meningioma may also arise in the ventricles, the location where chordoid gliomas occur (see Table 1). NHERF1 has a membranous expression in some of these neoplasms and an overall decreased cytoplasmic expression, but a distinctive microluminal expression as in chordoid meningioma was not observed, indicating that NHERF1 is useful in resolving the differential diagnosis with these chordoid and mucinous neoplasms.

In conclusion, our analysis of NHERF1 expression in meningioma uncovered (1) previously unknown elements of epithelial differentiation in chordoid meningioma that allow the differential diagnosis with other architecturally similar neoplasms and (2) an additional subset of high grade meningiomas with epithelial differentiation and sheeting, clear cell, rhabdoid and papillary-like morphology. This study provided a reliable marker for the epithelial differentiation of meningioma and further shed light on the vast heterogeneity of this neoplasm.

## MATERIALS AND METHODS

### Human specimens

Formalin-fixed paraffin-embedded brain tumor resection specimens were obtained from the authors’ institutions. The cases of chordoid meningioma, chordoma and chordoid glioma were retrieved retrospectively, as well as collected prospectively. The rest of the cases were collected prospectively and date from 2014 to 2017. All meningioma cases were graded according to the 2016 WHO Classification of Tumors of the CNS [1]. The morphological meningioma variants were diagnosed if more than 50% of the morphologic pattern was present in the tumor [14]. The WHO grade II meningiomas in this study show either increased mitotic count (more or equal to 4 mitotic Figures per 10 high power fields (HPFs) by visual inspection), brain invasion, or are high risk variants (chordoid and clear cell). The other risk features of meningioma, e.g. necrosis, may or may not be present. All WHO grade III meningiomas in this study have more than 20 mitotic Figures per 10 random HPFs. The demographics include the age and gender of the patients and the location of the tumor for all cases. These studies were performed in compliance with the ethical guidelines of the Helsinki Declaration and approved by the ethical committees for research on human subjects of the authors’ institutions.

### Histology, immunohistochemistry (IHC) and imaging

The specimens were processed for H&E staining and IHC as described [8, 9], with antibodies for NHERF1 1:3200 (Thermo/Fisher, Waltham, MA, USA), moesin 1:100 (3150, Cell Signaling Technology, Danvers, MA), NF2 C-terminal 1:800 (C18) (see also [35] (Santa Cruz Biotechnology, Santa Cruz, CA, USA) and EMA 1:400 (Dako, Carpinteria, CA) on a Leica automated IHC platform (Leica Biosystems, San Diego, CA, USA). The protocol for NHERF1 immunostaining was subsequently validated for clinical use according to the College of American Pathologists (CAP) laboratory quality assurance guidelines on a Ventana Benchmark Ultra platform (Roche/Ventana Medical Systems Inc., Tucson, AZ, USA) at 1:2000 dilution. Progesterone receptor (1E2) rabbit monoclonal antibodies (Roche/Ventana) IHC was used on selected cases. Cytology preparations were obtained from fresh specimens and processed by H&E manual staining during intraoperative diagnosis. Images were acquired at various magnifications with an Aperio Scanscope CS2 whole slide image system (Leica Biosystems) or with a Nikon Eclipse Ci microscope equipped with a Nikon Digital Sight DS-Fi2 camera (Nikon Instruments Inc., Melville, NY, USA), by using the Nikon NIS Elements 4.51.00 program.

### Transmission electron microscopy (TEM)

An approximately 8 mm^3^ tumor sample was processed for microvilli detection, as previously described [7, 36]. Briefly samples obtained either fresh, from prior overnight 10% formalin fixation or following deparaffination were fixed with 3% glutaraldehyde in 0.1 M sodium cacodylate buffer (pH 7.3) and postfixed with 2% osmium tetroxide. Samples were dehydrated in increasing concentrations of acetone, infiltrated, embedded in Spurr’s medium, and polymerized at 70°C overnight. Ultrathin sections were stained with either 3% uranyl acetate or UranyLess (Electron Microscopy Sciences, Hatfield, PA, USA) and lead citrate and examined in a Hitachi 7650 electron microscope (Hitachi High Technologies, Schaumburg, IL, USA). Digital images were obtained by using AMT Image System (Advanced Microscopy Techniques, Danvers, MA, USA).

### Statistics

The densitometric analysis was performed with the ImageJ program (National Institutes of Health, Bethesda, MD, USA) as previously described [37]. The NHERF1-labeled microlumen density was expressed as the mean of three counts in three contiguous 60× objective HPFs of a Nikon Eclipse Ci microscope performed at the highest microlumen density in the specimen. Numerical data were examined for normality of distribution and expressed as mean ± SEM, unless mentioned otherwise, by using the GraphPad Prism program (GraphPad Software, La Jolla, CA, USA). Differences between groups were assessed by using unpaired two-tailed *t*-test with or without Welch’s correction for variances significantly different and parametric data correlation was assessed by Pearson correlation coefficient, as described [38, 39]. Statistical significance was considered for *P* < 0.05. Confidence intervals for all tests were 95%.

## SUPPLEMENTARY MATERIALS FIGURES AND TABLE



## Abbreviations

CNS: central nervous system
EMA: epithelial membrane antigen
EMT: epithelial to mesenchymal transition
ERM: ezrin-radixin-moesin
H&E: hematoxylin eosin
HPF: high power field
NF2: neurofibromin 2 gene product (merlin)
NHERF1/EBP50: Na/H exchanger 3 regulatory factor 1/ERM-binding phosphoprotein 50
TEM: transmission electron microscopy
WHO: World Health Organization
