# Supplementary material for: Epithelial differentiation with microlumen formation in meningioma: diagnostic utility of NHERF1/EBP50 immunohistochemistry
Source: Oncotarget. 2018 Jun 19;9(47):28652–65. doi: 10.18632/oncotarget.25595 (PMC6033365; doi:10.18632/oncotarget.25595)
Supplement: Supplementary file 1 [file oncotarget-09-28652-s001.pdf]

Epithelial differentiation with microlumen formation in meningioma: diagnostic utility of NHERF1/EBP50 immunohistochemistry

SUPPLEMENTARY MATERIALS

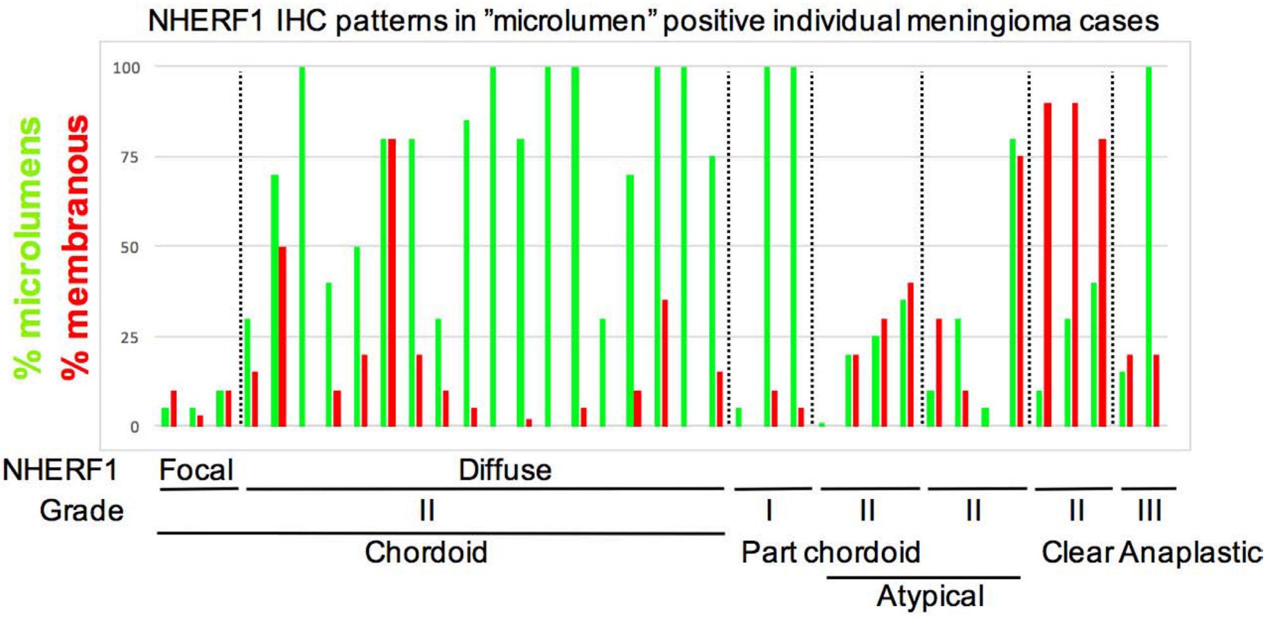

Supplementary Figure 1: NHERF1 IHC patterns in meningioma. A Contingency graph of NHERF1 IHC microlumen and membranous patterns, expressed as % of total tumor from a representative slide, in the individual cases of meningioma that showed microlumen-like NHERF1 staining. Note the high extent of microlumen positive areas in chordoid meningioma with relatively low extent of membranous staining, in contrast to the other atypical meningiomas that have relatively extensive membranous labeling.

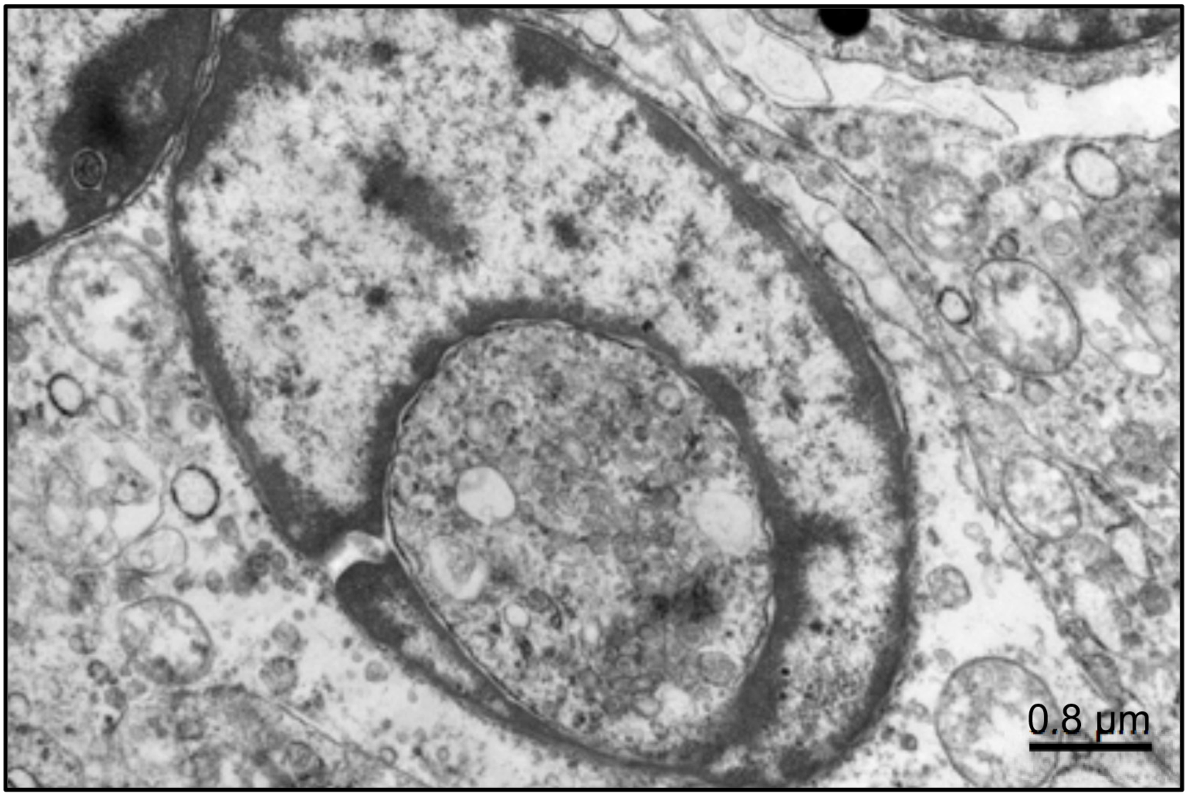

Supplementary Figure 2: Nuclear inclusion in secretory meningioma. A EM shows an intracytoplasmic lumen as intranuclear pseudoinclusion in a case of secretory meningioma.

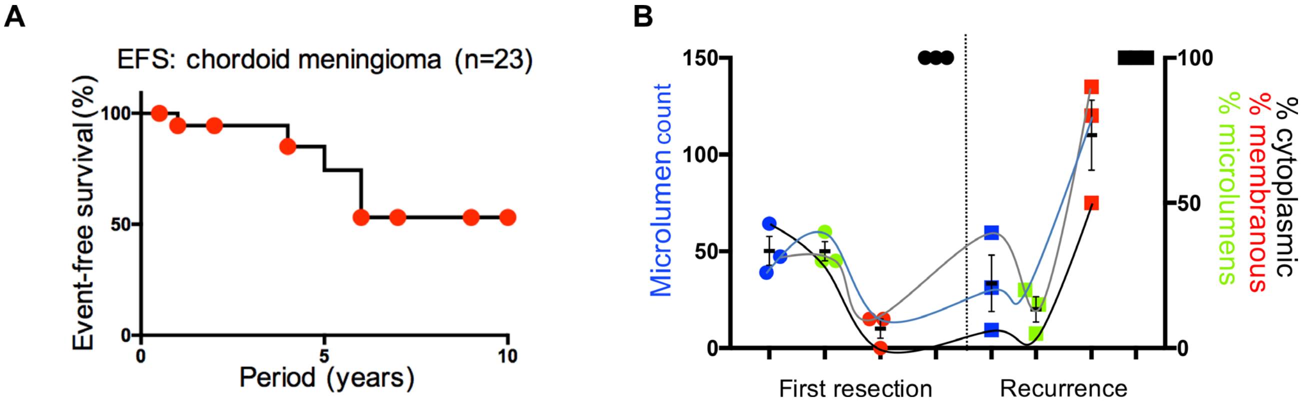

Supplementary Figure 3: Recurrence rate and NHERF1 patterns in chordoid meningioma. (**A**) Recurrence/event free survival of 23 cases of chordoid meningioma: 5 cases had recurrence, 3 were lost to follow-up shortly after surgery, 1 patient died of stroke postoperatory and 14 are currently in follow-up showing stable MRI. (**B**) Patterns of NHERF1 IHC in 3 recurrent chordoid meningioma cases, showing decrease in microlumens and increase in membranous NHERF1 expression in recurrent cases.

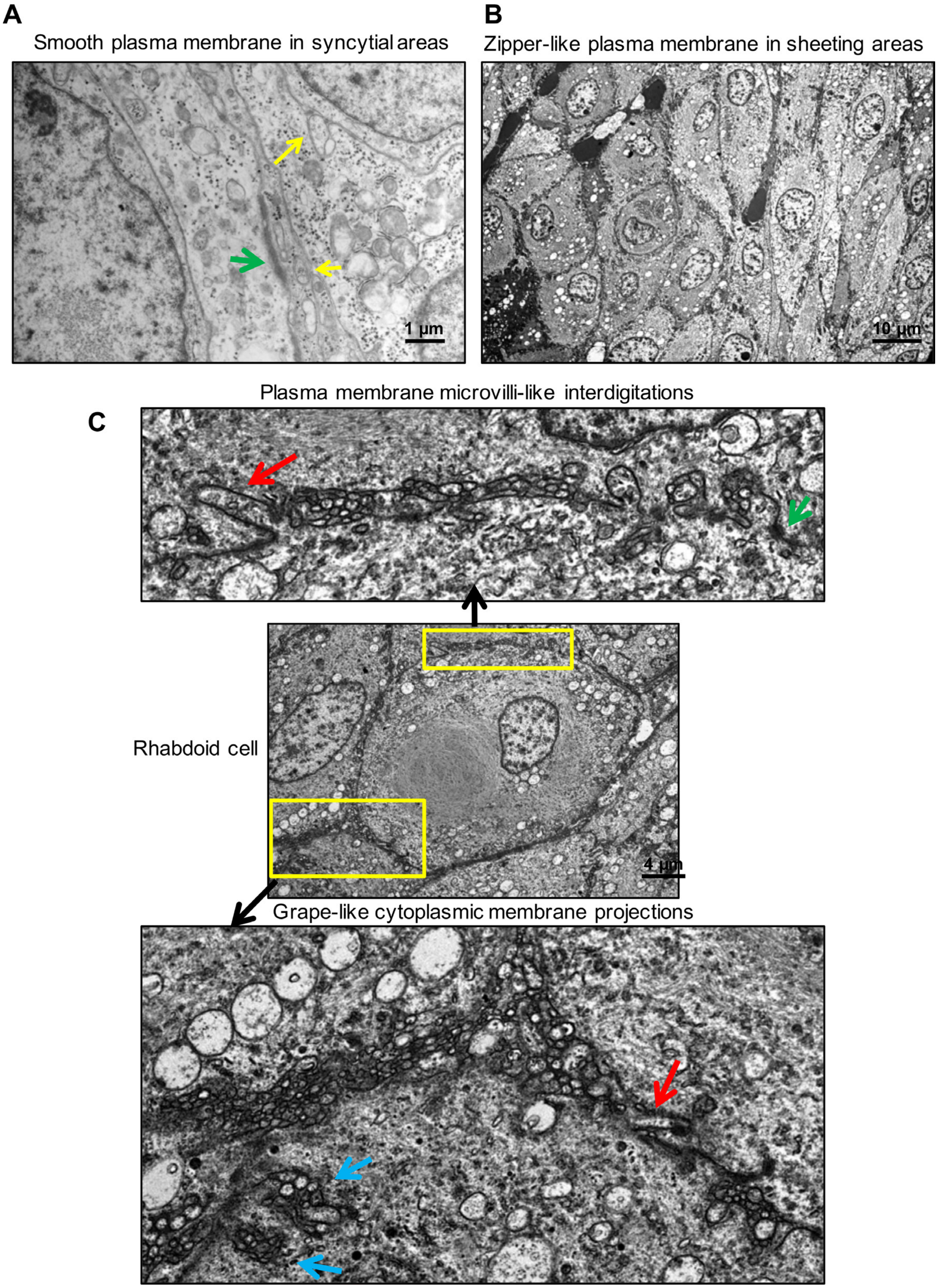

Supplementary Figure 4: Zipper-like plasma membrane in sheeting areas and rhabdoid meningioma. (**A**) EM of meningothelial meningioma shows smooth plasma membrane with loose interdigitations (yellow arrows) and intercellular junctions (green arrow). (**B**) Sheeting area in atypical meningioma shows tight zipper-like plasma membrane interdigitations. (**C**) Rhabdoid meningioma cell showing zipper-like plasma membrane formed by tightly juxtaposed microvilli-like interdigitations (red arrows), short intercellular junctions (green arrow – upper panel) and grape-like plasma membrane projections into the cytoplasm (blue arrows – lower panel).

Supplementary Table 1: Quantification of NHERF1 IHC patterns

Meningioma WHO grade	Case	Mean ML density	SD	ML pattern	Membranous	Cytoplasmic	Cytoplasmic intensity
Chordoid II	1	0	0	0	0	100	+
	2	0	0	0	0	100	+
	3	0	0	0	0	100	+
	4	0	0	0	0	100	+
	5	12	7.07	5	10	100	+
	6	13.67	2.62	5	3	100	+
	7	16.33	5.73	10	10	100	+
	8	7	1.41	30	15	100	+++
	9	19.33	3.09	70	50	100	+++
	10	36.67	12.81	100	0	100	+++
	11	39	5.71	40	10	100	+++
	rec	31.33	11.58	20	80	100	+++
	12	40	15.64	50	20	100	+++
	13	43.33	9.46	80	80	100	+++
	14	46	6.48	80	20	100	+++
	15	47.33	7.13	30	10	100	++
	rec	59.67	16.75	15	90	100	++
	16	55.67	15.69	85	5	100	+++
	17	59.67	7.93	100	0	100	+++
	18	61.33	9.53	80	2	100	+++
	19	64	7.87	100	0	100	+++
	20	64.33	3.091	100	5	100	+++
	21	64.33	16.43	30	0	100	+++
	rec	9.33	3.68	5	50	100	+++
	22	69	4.96	70	10	100	+++
	23	84	9.89	100	35	100	+++
	24	100	16.26	100	0	100	+++
	25	144	8.98	75	15	100	+++
Part-chordoid I	26	10	2.94	5	0	100	+
	27	37.33	3.68	100	10	100	+++
	28	109.33	17.96	100	5	100	+++
Part-chordoid II	29	10.33	1.24	1	0	100	+
	30	15.33	5.31	20	20	100	++
	31	21.33	8.37	25	30	100	++
	32	48.67	5.90	35	40	100	++
Atypical II	33	5.33	2.05	10	30	100	+

	34	35.33	5.24	30	10	100	++
	35	53	12.83	5	0	100	+
	36	69.67	4.71	80	75	100	+
Anaplastic III	37	0	0	0	0	0	sarcomatous
	38	27.33	11.02	15	20	30	+++
	39	75.67	12.91	100	20	100	+++

Abbreviations: ML, microlumen; SD, standard deviation; rec, recurrence.
